# Composition of the Putative Prepore Complex of *Bacillus thuringiensis* Cry1Ab Toxin

**DOI:** 10.4236/abc.2015.54014

**Published:** 2015-06

**Authors:** Manoj S. Nair, Donald H. Dean

**Affiliations:** 1 Department of Chemistry and Biochemistry, The Ohio State University, Columbus, OH, USA; 2 Aaron Diamond AIDS Research Center, Rockefeller University, New York, NY, USA

**Keywords:** Cry1Ab Protein, Protein-Receptor Interactions, Brush Border Membrane Vesicles, Small Unilamellar Vesicles, Prepore, LC-MS/MS

## Abstract

Prepore formation is hypothesized to be an obligate step in the insertion of Cry1Ab toxin into insect brush border membrane vesicles. We examined the architecture of the putative prepore when isolated using the published protocols [1] [2]. Our results demonstrate that the putative prepore form of Cry1Ab is a combination of receptor proteins attached to the toxin, when purified. The results also suggest that this prepore form as prepared by the methods published is different from other membrane-extracted oligomeric forms of Cry toxins and prepore of other toxins in general. While most other known prepores are composed of multimers of a single protein, the Cry1Ab prepore, as generated, is a protein-receptor complex oligomer and monomers of Cry toxins.

## 1. Introduction

Pore-forming toxins represent approximately 30% of bacterial toxins [3]. Highly opportunistic bacterial pathogens produce these toxins to perturb the rigid compartmentalization of plasma membrane by clustering into homo or hetero assemblies within the membrane. Even as pore formation is the main proposed pathogenicity determinant of these toxins, very little is resolved about this crucial step in the mechanism of action of these toxins [4]. This family of toxins has received considerable attention due to their widespread use as insecticides, more so in transgenic crops and the mechanism of action has been studied for more than two decades [5]–[8].

Most pore forming toxins including the Cry toxins are known to oligomerize during their mechanism of action. It is not clear whether the process begins with monomers of Cry toxins that dimerize and then add on monomers till a definite oligomeric shape is achieved or if monomers interact with preformed higher oligomers to form the channel structure. Bacterial toxins, in general, have been shown to form “prepore” oligomers that allow their insertion into target cell membranes. The term “prepore” refers to an intermediate state of these toxins in which the monomers of the toxin assemble to form a precursor that is competent to insert into these membranes. Many bacterial toxins that form prepore do undergo a substantial conformational change in the process, including a change in the secondary structure of the huge regions involved in the pore formation as in cytolysins [9] [10] and in anthrax toxins [11]. While receptor binding is either obligatory or enhances the formation of the prepore form in these toxins, the oligomers made from these toxins are composed majorly if not exclusively of the actual toxin that forms the pore [12] [13]. Oligomers made of exclusively Cry1Aa, Cry1Ab and Cry1Ac toxins have been isolated from the protease-treated brush border membrane vesicles in several studies [14] [15]. However, none of these studies examine the kinetics of formation of a prepore oligomer form of Cry1A toxins.

Cry1Ab toxin from *Bacillus thuringiensis* has been the model for studying prepore formation and oligomerization of Cry toxins [16]–[19]. In the prepore model, Cry1Ab as represented is a tetramer formed by the conformational change induced in the monomer upon binding cadherin receptor and losing alpha helix 1 that allows the oligomer to bind aminopeptidase N and alkaline phosphatase to mediate insertion of the toxin into the membrane [16] [20]. The conformational change was considered significant enough that the affinity of the cadherin receptor for Cry1Ab drastically reduced from the initial 1 nM affinity even as it was hypothesized to be obligatory for binding APN and alkaline phosphatase with high affinity [16] [21]. Given that the proportion of all the receptors for Cry protein was high on the insect cell surface, it was surprising that the Cry toxin was considered independent of the receptors as it bound or released from any of the receptors during pore formation. Another intriguing factor was that a polyclonal antibody designed to react with denatured Cry1Ab (potentially capable of interacting with multiple epitopes) would not react with the prepore. The aim of this study was to isolate the prepore as published [1] [2] for characterization of the mass of the toxin and to determine the regions of the monomer toxin that were retained in the prepore oligomer.

## 2. Materials and Methods

### 2.1. Source for Strains and Antibodies

*Bacillus thuringiensis sotto* (for Cry1Aa), *Bacillus thuringiensis* 4*Q*7 (acrystalliferous strain for expression of Cry1Ab F371C mutant) and *Bacillus thuringiensis HD*1–19 (for Cry1Ab) strains were obtained from *Bacillus* Genetic Stock Center, The Ohio State University. Antibodies used in this study include anti-Cry1Ab polyclonal antibody generated against denatured Cry1Ab monomer protein and anti-Cry1Ab polyclonal antibody kindly provided by Dr. Alejandra Bravo of Universidad Nacional Autónoma de México (which is referred to in this manuscript as the anti-prepore antibody). Antibody to the cadherin (anti Bt-R1) was kindly provided by Dr. Lee A. Bulla Jr. from University of Texas at Dallas.

### 2.2. Production of Prepore Oligomers

Prepore was produced by two separate methods that were published [1] [2]. Briefly, in the first method, 1 µg protoxin was mixed with 10 µg insect brush border membrane vesicles (BBMV) in the presence of 50 µl of solubilization buffer (50 mM Na_2_CO_3_ pH 10.5 + 0.2% β-mercaptoethanol) and incubated at room temperature (25°C – 28°C) for 15 min. In the second method, 200 ng of protoxin was incubated with scFv73 in a 1:2 mass ratio and 5% midgut juice was added in 100 µl of solubilization buffer containing 60 µM small unilamelar vesicles made of 1,2-Dioleoyl-sn-Glycero-3-phosphocholine (DOPC). The mixture was incubated at 37°C for 1 hr, followed by precipitating the membrane bound toxin at 400,000× g in a Beckman L8 ultracentrifuge. A control reaction, which lacked any small unilamellar vesicles, was used to show no precipitate formation in the absence of SUV. The pellet was resuspended in 50 µl of buffer in presence of 10% n-octyl-β-D-glucopyranoside and clarified by centrifugation, treated with loading dye and boiled for 3 – 5 min. Western blot analysis of the protein was performed using polyclonal anti-prepore antibody (1:50,000; 1hr) and secondary HRP antibody (1:10,000; 1hr) and was detected using chemiluminescence substrate (Bio-Rad).

### 2.3. Purification of the Prepore Oligomers

Cry1Ab prepore oligomers were prepared and purified using the two published methods [1] [2] using a Superdex 200 HR 16/60 (GE Healthcare) on an AKTA Explorer 100 (GE Healthcare). Several prepore samples were purified and pooled to yield 1 – 5 µg of sample. Sample volume of 2 ml was loaded into the column for every run. The flow rate was 1 ml/min and fractions of 2 ml were collected in a mobile phase containing protease inhibitors and 0.01% n-octyl-β-D-glucopyranoside. Purified proteins were analyzed using Western blots with polyclonal antibody prepared against prepore form of Cry1Ab at dilutions mentioned above. Purified samples isolated by method 1 were compared against buffer control in a Beckman XL-I analytical ultracentrifuge to obtain the closest molecular masses using sedimentation velocity measurements.

### 2.4. Mass Spectrometric Identification of Prepore Contents

In order to determine the identity of the proteins in the purified prepore complex, SDS-PAGE-resolved Cry1Ab prepore was subjected to LC-MS/MS analysis, performed at the W.M. Keck Facility, New Haven, CT. Briefly, purified prepore was run on SDS-PAGE gel and the entire lane from the gel, which showed cross-reactivity to the prepore antibody, was cut into 1 mm slices and digested with trypsin using in-gel digestion and the resulting samples were desalted in a 100 micron ID C18 column (Waters) in a gradient of 2% – 98% acetonitrile in the presence of 0.01% trifluoroacetic acid. Protein was considered identified if two or more peptides matched to the same protein accession numbers in the database (MASCOT analysis).

### 2.5. Toxicity Measurements of Isolated Prepore Form to Manduca Sexta Larva

Although the purified form of the prepore had a mixture of oligomer and monomer forms, we nevertheless performed limited toxicity bioassays on them to measure any improvement over the purified monomer toxins as reported. Toxicity levels were determined by estimating the median lethal concentration (LC_50_) on first instar *M. sexta* larvae using the diet surface contamination assay [22]. Sixteen first instar larvae were used for each concentration of the toxin, and a total of six concentrations of each toxin were used. Mortalities were recorded after 5 days. The LC_50_ for each toxin was calculated by Probit analysis using SoftTox (WindowsChem Software, Inc.).

## 3. Results

### 3.1. Formation and Purification of Prepore Complex

**Figure 1:**
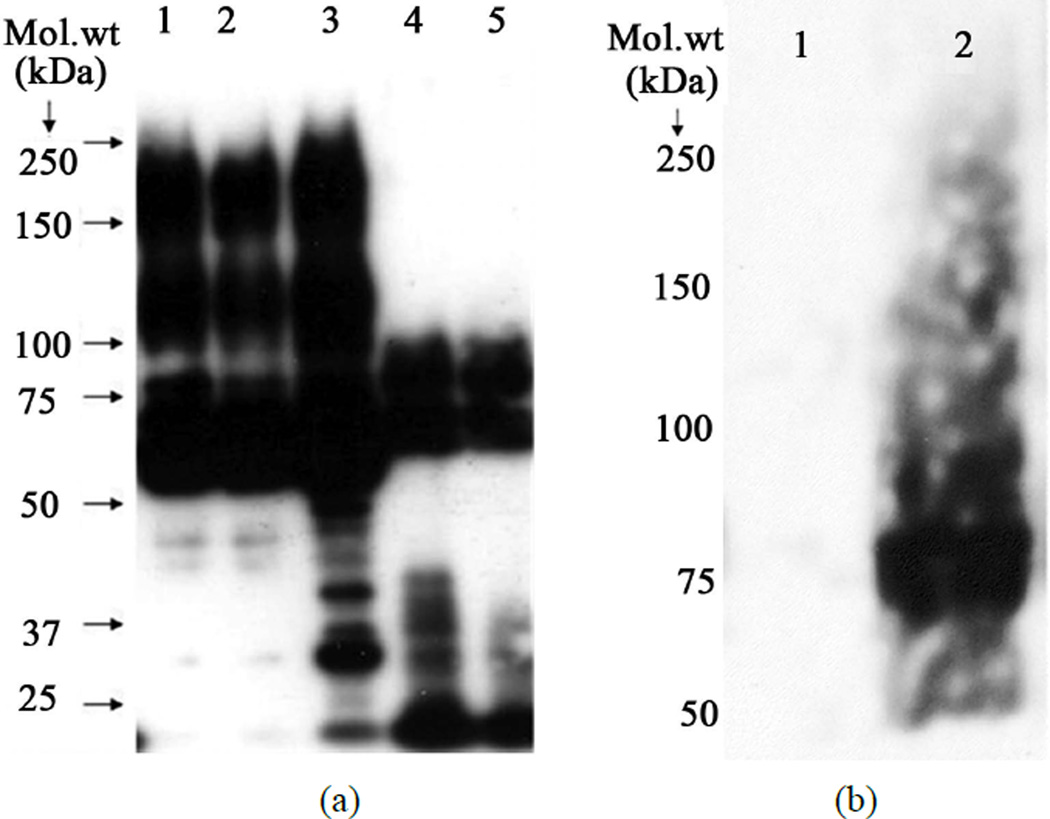
(a) Detection of prepore from Cry toxins using anti polyclonal antisera obtained from Bravo, A, *et al*. [1] at 1:50,000 dilution. Lane 1 = Prepore from Cry1Abwt produced in *Bt*HD1–19. Lane 2 = Prepore from Cry1Ab F371C mutant produced in *Bt*4Q7. Lane 3 = Prepore from Cry1Aa wt produced in *Bt sotto* 4E3. Lane 4 = Cry1Ab produced in *E. coli*. Lane 5 = Cry1Aa produced in *E. coli*; (b) Detection of cadherin association in purified prepore of Cry1Ab. Monomer and prepore obtained were treated with anti Bt-R1 antibody at 1:10,000 dilution of the primary antibody. Lane 1 = Purified Cry1Ab monomer toxin (200 ng), Lane 2 = Purified Cry1Ab prepore toxin (50 ng).

**Figure 2:**
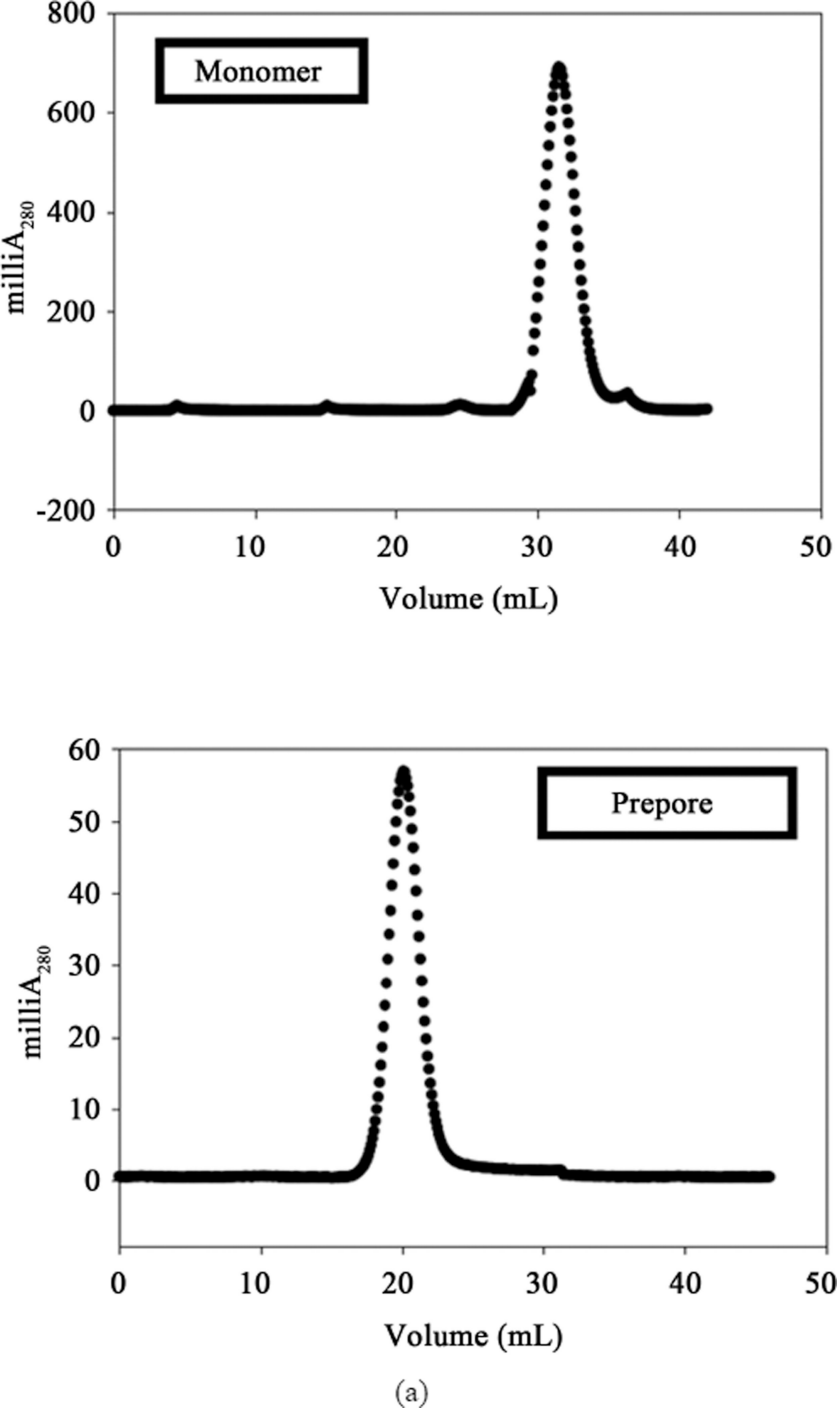

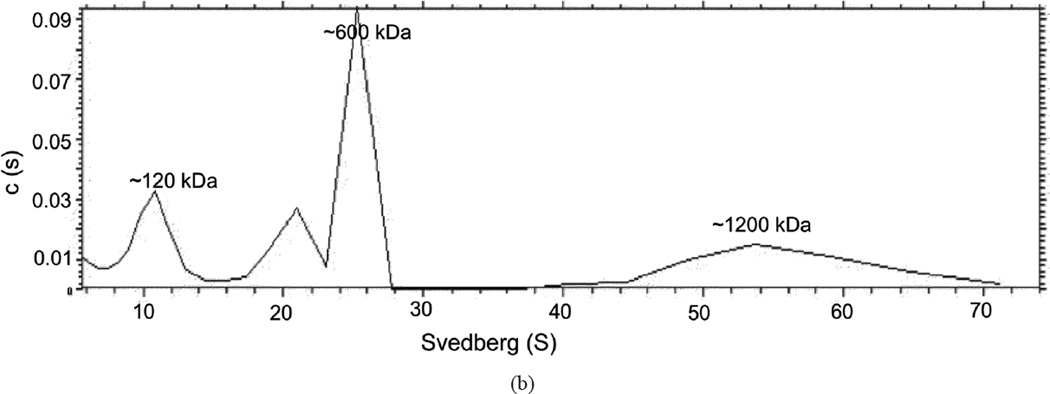
(a) Superdex 200 HR 16//60 gel filtration column purification of the prepore complex. The two chromatograms show the elution profile of monomer toxin versus the prepore complex as indicated; (b) Sed-Fit analysis of sedimentation velocity measurements in a Beckman XL-I analytical ultracentrifuge of prepore sample purified from Superdex 200 HR column. Molecular weights indicated on each peak were deciphered using a Nomogram to an approximation.

Using the published protocols [1] [2] of prepore discovery, we examined oligomer formation of Cry1Aa and Cry1Ab either with BBMV or with scFv73 in the presence of lipid vesicles. Notably, while our Cry1Ab polyclonal antibody raised against denatured monomer toxin did not recognize any oligomer product on western blots (data not shown), the polyclonal prepore antibody we received showed considerable amounts of prepore for both Cry1Aa and Cry1Ab, while also showing equal or higher amounts of monomers of the toxin on these blots (Figure 1(a)). Based on the hypothesis that receptor interaction may have modified the protein to form oligomers, we decided to measure the reactivity of the Cry1Ab to anti-Bt-R1 antibody after production of the prepore. We purified the prepore made using the BBMV-based method [1] by Superdex 200 gel filtration column as one single peak (Figure 2(a)) and tested the sample on western blot against the cadherin antibody. The blot indicated presence of a 75 kDa predominant band that was not present in the monomer form of Cry1Ab (Figure 1(b)). The purified prepore when subjected to sedimentation velocity measurements indicated the presence of multiple peaks suggestive of molecular complexes of different sizes (SedFit analysis) as indicated in Figure 2(b).

### 3.2. Mass Spectrometry Analysis of Prepore of Cry1Ab

Protein samples verified for formation of the prepore form were purified in order to determine the regions of Cry1Ab present/absent in the prepore. Since SDS-PAGE of purified prepore showed multiple bands, we decided to characterize each band to determine toxin regions present in the prepore form and its variations if any, from the monomer. Proteins were identified from in-gel trypsin digests of the SDS-PAGE bands using LC MS/MS analysis of the digested bands. Proteins were identified from a MASCOT search with two criteria: two or more MS/MS spectra match the same protein in the database and that each of the matched peptides was ensured to be from trypsin digestion.

Prepore examined from the two methods showed presence of Cry1Ab toxin. However, the purified prepore in both the methods had additional proteins that were identified by the sensitive MS/MS process. The entire list of pertinent proteins identified is listed in Table 1. Prominent candidates include insect cadherin and aminopeptidase in the prepore that was produced in the BBMV-based method [1] and peptides homologous to the single chain antibody (scFv73) in the method that was based on using lipid vesicles [2].

**Figure 3:**
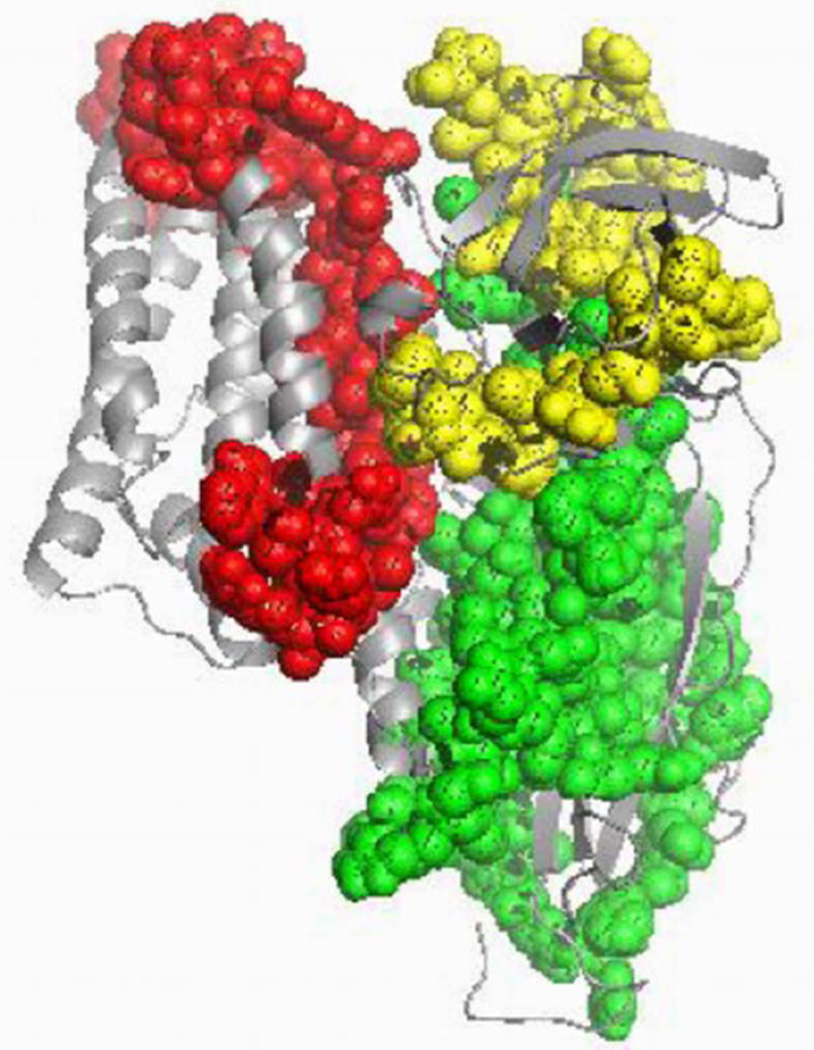
Regions of Cry1Ab that were identified based on peptides matched from LC-MS/MS of digested Cry1Ab prepore samples. Red color indicates peptides matched to Domain I residues, green color indicates peptides matched to Domain II residues and yellow color indicates peptides matched to Domain III residues.

Of interest was the observation [2] [23] that presence of small unilamellar vesicles allowed prepore form of the protein to be inserted into the membrane and that the prepore form was collected by ultracentrifugation of the vesicles. Since models of insertion have predicted alpha helix 4 and 5 of domain I to be involved in membrane insertion, we were curious to determine the regions of prepore embedded in the artificial membrane in the presence of the cadherin-like peptide (scFv73). The prepore of Cry1Ab was extracted out of the lipid vesicles using n-octyl-β-D-glucopyranoside, and the regions of the toxin that were present were identified from the peptides that were detected in the LC-MS/MS. As shown in Table 2, alpha helices 5, 6, and 7 of domain I was presented in the membranes. However, more peptides were identified from domain II and domain III of the prepore form of the toxin. The regions of Cry1Ab to which the peptides were mapped have been highlighted (Figure 3) with differential coloring for each domain, indicating presence of all three domains of the Cry1Ab toxin in the putative prepore isolated.

### 3.3. Toxicity Measurements of Monomer and Prepore Forms of Proteins

Even though we could not quantitate the amount of prepore form of Cry1Ab in the reaction mixture due to interference of monomers in all methods, we decided to perform a comparison of toxicity measurements of prepore as isolated by the published procedures to the pure monomer based on the premise that the conformational change would enhance the toxicity of Cry1Ab protein due to the presence of the active intermediate. Our studies using diet surface contamination assays with *Manduca sexta* first instar larvae showed overlapping LC_50_ confidence limits for prepore and monomer as listed in Table 3.

## 4. Discussion

The putative prepore formation of Cry1Ab toxin was reproduced using published protocols [1] [2] and materials provided by the Bravo group who pioneered the serial-receptor binding model. The goal of this study was to examine the resultant form using proteomic and analytical methods. The oligomer form of the toxin was generated in the presence of the cadherin receptor and/or the scFv73 peptide that mimicked the cadherin receptor. Purification of the prepore complex via molecular sieving resulted in a single peak albeit one that eluted at a molecular weight of >250 kDa through a Superdex 200 size exclusion chromatography column (*i.e.* beyond the void volume). This peak reacted to polyclonal Cry1Ab antibody on western blot from a reducing SDS-PAGE gel run as a tetramer form. However, the SDS-PAGE of the single peak generated smaller bands of monomer toxin and other proteins, certainly ones that were associated with the toxin in the putative prepore form as prepared. Sedimentation velocity measurements of the purified oligomer indicated that the native forms of the toxin persisted in many sizes ranging from monomer to dodecamer as deciphered by the limits of nomogram analysis. To test if only a limited portion of the toxin was associated with the membrane in the prepore as predicted by models, we extracted the membrane inserted form of prepore complex obtained by using scFv73 peptide with a detergent. LC-MS analysis of the resulting tryptic digest indicated presence of peptides from all domains of the Cry1Ab in the extracted form. It also indicated presence of scFv73 peptide or the receptors depending on the method of prepore formation that was pursued. Toxicity measurements of the Superdex 200 column purified oligomer did not provide a LC_50_ that was any significantly higher than the monomer form.

We now discuss our observation that the putative prepore does not react against a polyclonal antibody formed against denatured Cry1Ab. The possibility that a conformational change would obviate binding of the polyclonal antibody exists but seems unlikely since the antibody binds to both native and denatured forms of the toxin. We suggest that the Cry1Ab toxin in the putative prepore is masked by the scFv73 peptide or receptors to the extent of blocking it from interaction to the anti-Cry1Ab antibody. In addition, the anti-prepore antibody, which does bind to the putative prepore is observed to bind to purified cadherin (data not shown).

All of these data in combination suggest that while putative prepore forms of Cry1Ab can be successfully generated, the resultant product as examined is not a pure toxin. Since very low amounts of toxin are effective at mediating toxicity, based on the hypothesis of the serial receptor binding model [16], it is likely that a very small fraction of oligomer may mediate most of the toxicity. However, our LC_50_ analysis of the purified product does not show significant variation in overall toxicity between monomer and produced oligomer forms. To be more precise, the contribution of each form to the overall toxicity is not accounted by the procedures used [1] [2] to isolate and purify the putative prepore toxin. Based on the data, the monomer appears to account for most of the toxicity in solution, suggesting a possibility of oligomers of Cry toxin being formed only post-insertion in the presence of the membrane bilayers.

The current data also questions the nature of the membrane bound tetramer as proposed by the serial receptor binding model, which indicates that only two alpha helices of the toxin from each monomer are present within the membrane. The rest of the toxin, if not associated with the membrane, should be easily digested by proteases. Our tryptic digests of membrane extracted prepore isolates presented almost equal number of peptides from all three domains of active Cry1Ab toxin (5 from Domain I, 7 from Domain II and 5 from Domain III) and none from the C-terminal region of the protoxin form used in these prepore formation methods or any peptides matching alpha helix 1 of active toxin. In addition, there are receptor peptides that were bound to the toxin. Our previous studies also highlight an intact 60 kDa monomer toxin that lacks alpha helix 1 as the form that inserts in the membrane [24]. The cumulative results suggest that the prepore form we isolated and purified is a form of the *Bt*-booster of Cry toxins that has been successfully isolated at higher concentrations of receptors and has shown to have higher toxicity than the toxin alone [25]. The verity of the serial receptor binding model has also been challenged by other investigators investigating the mechanism of toxin induced cytotoxicity via a Bt-R1 mediated adenyl cyclase-protein kinase A signaling pathway [26] [27]. In fact, the authors of the serial-receptor binding model have since modified their view of the mechanism [20] indicating the deficiencies of their model as proposed [16].

In summary, we performed a proteomic and biophysical analysis of the putative prepore of Cry1Ab to determine the size and the contents of the complex and have discovered that the toxin does exist as a higher order tetramer as the serial-receptor model hypothesizes but not as a pure toxin protein; it is complexed with receptor proteins and forms several higher order species besides the tetramer. We conclude that the putative prepore of Cry proteins is not a classic prepore as proposed for other protein toxins and should be referred to as a “complex oligomer” of Cry proteins and receptor proteins used to generate it. We did not detect alpha-helix 1 in the proteomic analysis of the complex oligomer, so it remains to directly determine if it is proteolytically removed upon interaction with the receptor or later upon insertion into the membrane. Our efforts toward resolving the structure of the prepore complex will address further details on the exact nature of the complex oligomer. From these studies, we question the exact role of a prepore generated using protocols described [1] [2] as an intermediate in the mode of action of the Cry toxins and suggest technical artifacts that need addressed before referring to the moiety as a typical prepore toxin.

## Figures and Tables

**Table 1:** Major proteins of known function isolated from LC-MS/MS from prepore purified from (a) BBMV-based method or (b) small unilamellar vesicle method of obtaining prepore.

(a)		

GI accession number of match	Protein identified	Maximum number of peptides identified
**gi|40255**	Insecticidal crystal protein	13
**gi|143099**	Insecticidal crystal protein	13
**gi|20465244**	Cadherin from *M. sexta*	5
**gi|2499901**	APN-like protein (Membrane alanyl aminopeptidase precursor Cry1Ac receptor)	32
**gi|8488965**	Aminopeptidase 2	24
**gi|20279109**	Aminopeptidase 3	18

(b)		

GI accession number of match	Protein identified	Maximum number of peptides identified
**gi|40255**	Insecticidal crystal protein	21
**gi|143099**	Insecticidal crystal protein	16
**gi|1902832**	Single chain (scFv) antibody Mol.wt. 26267	3
**gi|106429**	Ig heavy chain V region (alpha-phOx15)-human fragment Mol.wt. 13696	2

**Table 2:** Sequence of major peptides matched to Cry1Ab protein obtained from the LC-MS/MS of prepore sample isolated from detergent solubilization of small unilamellar vesicles of Cry1Ab and purified by gel filtration. The order of the peptides in the table is in decreasing order of the number of hits obtained from MASCOT.

Sequence of Peptides identified	Region of Toxin	Residue positions on toxin
DVSVFGQR	Domain I (alpha 5)	D174-R181
TLSSTLYR	Domain II (beta)	T361-R368
LSHVSMFR	Domain II (beta)	L430-R437
WYNTGLER	Domain I (loop between alpha 6 & 7)	W210-R217
TSPGQISTL	Domain III	T502-R511
GSAQGIEGSIR	Domain II	G282-R292
GPGFTGGDILR	Domain III	G490-R500
VNITAPLSQR	Domain III	V512-R521
IVAQLGQGVYR	Domain II	I350-R360
WGFDAATINSR	Domain I (loop between alpha 5 & 6)	W182-R192
EWEADPTNPALR	Domain I (loop between alpha 3 & 4)	E116-R127
EIYTNPVLENFDGSFR	Domain II	E266-R281
IEFVPAEVTFEAEYDLER	Domain III end (C-term of active toxin)	I602-R619
LEGLSNLYQIYAESFR	Domain I	L100-R115
ELTLTVLDIVSLFPNYDSR	Domain I (alpha 7)	E235-R253
SAEFNNIIPSSQITQIPLTK	Domain III	S458-K477
SPHLMDILNSITIYTDAHR	Domain II	S293-R312

**Table 3:** Toxicity measurements of monomer and prepore forms of Cry1Ab as measured by LC_50_ of toxin on *Manduca sexta* larvae.

Cry1Ab monomer	20.0 (7.5 – 31.7)
Cry1Ab oligomers formed in solution	28.0 (10.0 – 46.5)
Cry1Ab toxin extracted from membrane**	25.0 (5.0 – 35.0)
Cry1Aa prepore oligomer	17.1 (6.2 – 35.3)
Cry1Ab prepore oligomer	26.2 (3.9 – 40.8)
Cry1Ab F371C prepore oligomer	>2000

** Cry1Ab toxin in this group was obtained by dissolving the BBMV after insertion of toxin using 10 mM HEPES pH 7.5 buffer + 1%β-octyl glucoside detergent.
